# The Effect of Femoral Cutting Guide Design Improvements for Patient-Specific Instruments

**DOI:** 10.1155/2015/978686

**Published:** 2015-12-31

**Authors:** Oh-Ryong Kwon, Kyoung-Tak Kang, Juhyun Son, Yun-Jin Choi, Dong-Suk Suh, Yong-Gon Koh

**Affiliations:** ^1^Joint Reconstruction Center, Department of Orthopaedic Surgery, Yonsei Sarang Hospital, 10 Hyoryeong-ro, Seocho-gu, Seoul 06698, Republic of Korea; ^2^Department of Mechanical Engineering, Yonsei University, 50 Yonsei-ro, Seodaemun-gu, Seoul 03722, Republic of Korea

## Abstract

Although the application of patient-specific instruments (PSI) for total knee arthroplasty (TKA) increases the cost of the surgical procedure, PSI may reduce operative time and improve implant alignment, which could reduce the number of revision surgeries. We report our experience with TKA using PSI techniques in 120 patients from March to December 2014. PSI for TKA were created from data provided by computed tomography (CT) scans or magnetic resonance imaging (MRI); which imaging technology is more reliable for the PSI technique remains unclear. In the first 20 patients, the accuracy of bone resection and PSI stability were compared between CT and MRI scans with presurgical results as a reference; MRI produced better results. In the second and third groups, each with 50 patients, the results of bone resection and stability were compared in MRI scans with respect to the quality of scanning due to motion artifacts and experienced know-how in PSI design, respectively. The optimized femoral cutting guide design for PSI showed the closest outcomes in bone resection and PSI stability with presurgical data. It is expected that this design could be a reasonable guideline in PSI.

## 1. Introduction

Total knee arthroplasty (TKA) has high success rates with the majority of patients experiencing rapid improvement in pain, function, and quality of life [1]. Accurate bone resection and well-calibrated alignment are key factors in the success of TKA. As a favorable alternative to the standard procedure and navigation, PSI have been introduced recently as a means of improving bone resection accuracy through custom cutting blocks constructed using preoperative 3-dimensional (3D) imaging [2, 3]. Computer models of the distal femur and proximal tibia are defined from computer tomography (CT) or magnetic resonance imaging (MRI). Based on these models, presurgical TKA planning is performed and is available to the surgeon via web-based interfaces. These guides are used to manufacture cutting blocks that are precisely molded to the patients' anatomy and designed to reproduce the functionality of off-the-shelf conventional instruments. Both surgeons and manufacturers alike have suggested that the operative time can be reduced with the elimination of conventional instruments, which may translate into decreased costs and increased volume capacity for the surgeon. Furthermore, improvements in the accuracy of alignment and reductions in operative time have been suggested with PSI use [4].

These theoretical advantages have been postulated but have not been confirmed in the literature to date [5]. It is also unclear whether PSI improve TKA operation prognosis compared to the primary technique using conventional instruments [6]. Furthermore, concerning the PSI surgical technique, there are still varying opinions among orthopaedic surgeons regarding the relative advantages and disadvantages of CT versus MRI [7–9]. White et al. reported that MRI leads to higher costs, whereas CT is likely to provide the optimal surgical outcome at lower cost when it is used to manufacture patient-specific templates [7]. Asada et al. stated that both CT and MRI reduce operative time with the same accuracy in three planes, but MRI is not as cost effective [8]. Silva et al. implied that MRI may be more accurate than CT using the Signature system (Biomet, Inc., Warsaw, IN, USA) when planning surgical guides for TKA [9]. In order to provide PSI with the highest accuracy, 3D reconstructed images must be composed well in CT and MRI images and PSI should be fitted securely to the patient's anatomy. If the guide is not fitted securely, PSI stability is reduced and bone cutting is not completed as planned in the presurgical program, leading to malalignment. However, there have been no studies related to design improvements in PSI femoral cutting guides with respect to the quality of imaging or difference of individual anatomy.

Therefore, the purpose of this study is (1) to evaluate the effect of PSI design in CT and MRI with respect to secure fit and bone cutting, (2) to compare bone cutting in different PSI designs with respect to motion artifacts in MRI, (3) to compare bone cutting with respect to optimized PSI designs, and (4) to develop a preoperative plan and resultant custom guides that could accurately replicate surgeon preference with infrequent intraoperative changes.

## 2. Materials and Methods

### 2.1. Patient Enrollment

This retrospective cohort study was approved by the institutional review board of our hospital. All patients provided informed consent before surgery. Between March and December 2014, we included 120 patients with end-stage knee osteoarthritis scheduled for TKA in the study. Patients with rheumatoid arthritis, previous osteotomy, fractures, retained hardware in the limb, or claustrophobia were excluded. The inclusion criteria were diagnosis of primary knee osteoarthritis and the ability to undergo MRI at our facility. Mean patient age was 70.9 years (range, 64–85 years) and mean body mass index (BMI) was 27.4 kg/m^2^ (range, 20–42 kg/m^2^).

### 2.2. Image Protocol

For the 20 patients in the first group, both CT and MRI (1 mm and 2 mm slice thickness) images were taken for accuracy comparisons. CT images of the knee joint were taken with a slice interval of 1 mm using a 64-channel CT scanner (Somatom Sensation 64; Siemens Healthcare, Erlnagen, Germany), whereas 5 mm slice thickness was applied to hip and ankle joints. The tube parameters were 120 kVp and 135 mA. The acquisition matrix was 512 × 512. The field of view was 200 mm.

MRI images were acquired using a 1.5T MRI scanner (Achieva 1.5T; Philips Healthcare, Netherlands). MRI scans of the tibiofemoral knee joint were obtained at 1 or 2 mm slice thickness in the sagittal plane, whereas 5 mm slice thickness was applied to hip and ankle joints in the axial plane. For the nonfat saturation condition, MRI consisted of an axial proton-density (PD) sequence. A high-resolution setting was used for the spectral presaturation inversion recovery sequence (TE: 25.0 ms, TR: 3,590.8 ms, acquisition matrix: 512 × 512 pixels, NEX: 2.0, and field of view: 140 × 140 mm). All procedures were identical to those in Signature from Biomet.

### 2.3. Presurgical TKA Techniques and PSI Design Methods

The first author (Oh-Ryong Kwon) had participated in 30 TKA operative cases with Signature at the time of the study. Signature charges $900 to fit each patient's unique anatomy and to guide surgical bone resection in order to manufacture femoral and tibial PSI guides [10]. The time elapsed from the submission of the MRI to the receipt of guides was 4 to 6 weeks. However, it is illegal to impose charges related to surgical instruments in the Republic of Korea; thus our hospital has developed our own presurgical planning and design platform in order to provide PSI services to patients for free.

3D data can be acquired through either MRI or CT. The 3D reconstruction processes were performed with Mimics software (version 17.0; Materialise, Leuven, Belgium). Using Mimics, the resulting 3D images were converted to STL files and implemented in the digital CAD software, 3-Matic, also produced by Materialise. 3-Matic allows the user to combine geometry from mixed sources into a single project. PSI guides were designed with 3-Matic commercial software (version 9.0; Materialise, Leuven, Belgium).

**Figure 1:**
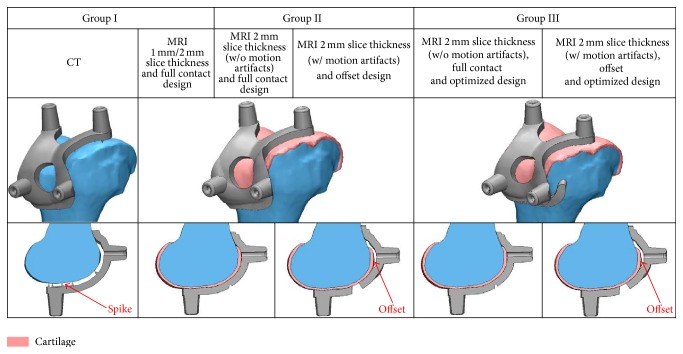
PSI guides of each group used in this study.

**Figure 2:**
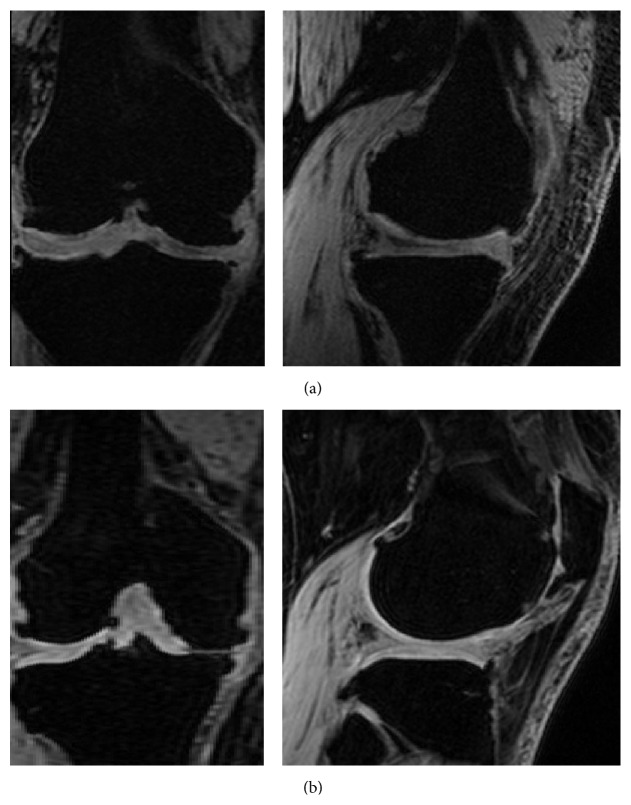
MRI images categorized based on the quality of images: (a) with motion artifacts; (b) without motion artifacts.

**Figure 3:**
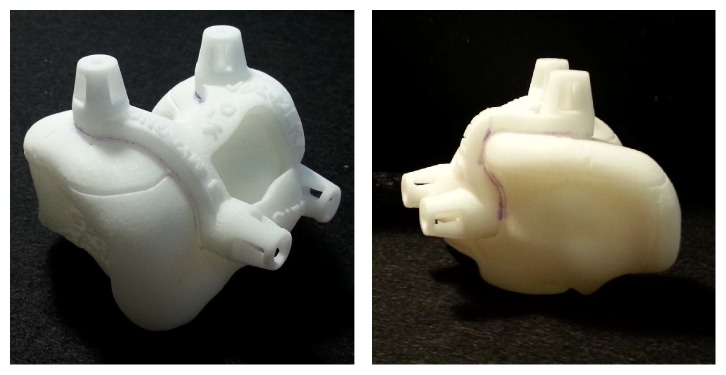
Signature system (Biomet, Inc., Warsaw, IN, USA) PSI guide for femur.

Patients from the first group were divided into two different groups with respect to slice thickness, 1 mm from CT, 1 mm from MRI, and 2 mm from MRI scans (Materialise). CT has limitations in delineating articular cartilage (3D model inaccuracies) [11, 12]. Cartilage was not able to be reconstructed using CT and 2 mm thickness was considered followed by contact with bone using spikes, in order to overcome the limitations of the apparatus (Figure 1). We believe that the more segmented the scans obtained, the more accurate the 3D model that could be developed from the images. PSI guides for MRI scans with 1 mm and 2 mm slice thickness in group 1 were designed with full contact. Following the analysis of the first group, we created the second group. For group 2, MRI with 2 mm slice thickness was used. Group 2 was categorized based on the quality of images (Figure 2). There are two designs with and without motion artifacts. A PSI design with only bone contact regions was constructed for scans with motion artifacts, whereas the tolerances were considered for others. For those without motion artifacts, a full contact design was developed according to the bony geometry (Figure 1). Throughout the learning curve, group 3 represented those with optimized designs. Group 3 was also divided into subgroups with respect to the patients' motion. However, group 3 is not only different in terms of motion artifacts; but they also had the advantage of preventing movement while drilling by using a perfect fit between bone and cutting guides (Figure 1). For the PSI design, each company has unique design techniques to make the bone fit guides (Figure 3). Our PSI design was optimized to be perfectly fitted to the bone at the anterior flange.

Each step was evaluated for each group and intraoperative changes were recorded, including resection level, component size, and coronal/sagittal alignment. The resected bone was then measured with a 3D laser scanner (Comet VZ; Steinbichler Optotechnik GmbH, Neubeuern, Germany) with 50 *μ*m accuracy. The distal femur resections were measured medially and laterally, thus obtaining distal femur medial and lateral resection measurements. Posterior femoral condyle cuts were made and resections in the medial and lateral posterior femoral condyles were measured. Bone cutting data was comparatively analyzed with preplanning results. The thickness of the saw blade was added to the resection thickness to calculate the total resection for each cut.

All operations followed by TKA surgical preplanning were conducted by an experienced surgeon (Oh-Ryong Kwon). A computer-generated preoperative plan was created according to the surgeon's preferences, as follows: default alignment for femoral component rotation was parallel to the surgical epicondylar axis, femoral component coronal alignment 90 degrees to the mechanical axis, and femoral component sagittal alignment 3 degrees of flexion with 9.5 mm distal medial resection. We retained the default plan when it appeared appropriate and recorded all changes when made. The time from submission of the 3D image to receipt of the guides was 4 days. All patients in both cohorts received a posterior-stabilized, fixed-bearing implant. The operation was performed through an anteromedial parapatellar approach, without everting the patella. Cement fixation was used in all patients. The implant used was the Genesis II Total Knee System (Smith & Nephew, Inc., Memphis, TN, USA).

The results presented are the intraoperative changes for measurements of femoral component size and alignment in groups 1, 2, and 3. The differences between preplanning results and actual bone cutting were measured in groups 2 and 3.

## 3. Results

One hundred and twenty patients underwent an operation with custom-fit technology.  Table 1 summarizes the demographic profiles. There were no hematomas, infections, manipulations, or reoperations.

Intraoperative changes to the implant sizing and alignment proposed by PSI were observed (groups 1–3). A total of 94 intraoperative changes were made in 120 TKAs (0.8 changes per knee) with the use of PSI (groups 1–3, Table 2). PSI predicted the implanted component size in 90% (*n* = 12) of femurs. PSI predicted the varus and valgus alignment and internal and external rotations in 96% (*n* = 5) and 89% (*n* = 13), respectively, of femurs.

Throughout the path from group 1 to group 3, the frequency of intraoperative changes of the implant size and alignment decreased. In group 3, the percentage of changes was 3% (*n* = 3). In group 1, for the 13 patients within the subgroup with 1 mm slice thickness MRI, the time spent in the MRI machine was long and impatience led to motion artifacts. Therefore, bone models and PSI guides could not be developed and manufactured due to the inaccuracy of the scans. In group 1, the PSI cutting guides that were manufactured using 1 mm slice thickness MRI scans were used for only one of 20 patients. Similarly, in group 1, the PSI cutting guides based on CT scans and 2 mm slice thickness MRI scans were used for four and fifteen patients, respectively.

The mean (±standard deviation) discrepancies between the predicted and actual resection thicknesses (“cutting error”) are shown in Table 3 (groups 2, 3). Those of groups 2 and 3 were 0.44 mm and 0.21 mm on the medial sides and 0.32 mm and 0.13 mm on the lateral sides, respectively. There was no significant difference between the two groups, but the femoral distal resection error diminished going from group 2 to group 3. The range of differences between the planned and the measured resections was larger for the medial posterior condyle resection, while the lateral posterior condyle resections were well matched with the planned results (groups 2, 3). The discrepancies between the planned and the measured bone resections were close to 0% in group 3. The difference between the planned and the actual bone resections was the lowest in the optimized design from group 3 without motion artifacts, and the values from group 3 with motion artifacts and group 2 without motion artifacts were similar. In other words, the superiority of an optimum PSI design has been proven.

## 4. Discussion

The most important finding of this study was the optimal medical imaging method and critical thickness for MRI slices for the design of PSI and the development of the optimal design for a PSI guide well-fitted to the patient's anatomy without any micromotion in contact with the bony surface.

The outcome of TKA greatly depends on the surgical technique used [13, 14]. Technical errors such as malalignment may lead to early failure [15]. Variations in surgical performance and outliers in TKA still occur and they may affect outcome. Improvement in the outcomes of TKA, particularly for difficult cases in young, active patients, patients with bone abnormalities, and patients who have revision surgery, remains a major concern [14]. Outcome improvement has to be viewed for cost-effectiveness and minimizing complications. Computer-assisted surgery aims to improve the alignment of the TKA components. However, all computer-navigated and robotic systems require an additional stage of registration, which can be time-consuming and costly; this is of particular concern in low-volume hospitals [6, 16, 17]. Despite the increased intraoperative complexity, initial reports on computer-assisted surgeries have been encouraging, but registry data show that conventional instrumented surgery remains the standard treatment [18]. PSI, built according to 3D medical images, have been designed to address the disadvantages of conventional techniques. PSI should be considered as an alternative to conventional instrumentation, but controversy remains regarding this issue [2, 4–6, 19]. The points of dispute are whether PSI improve tibiofemoral alignment [5, 6] and reduce blood loss due to shortened operation times compared to conventional methods [20]. There are also conflicting opinions on whether CT and MRI scans are more reliable for PSI [7–9].

We hypothesized, based on the experience using Signature PSI systems, that inaccuracy in 3D models and PSI guides not perfectly fitted to bone are the main two reasons for the resulting dissimilarities between preplanned outputs and measured bone resections in the operating room.

It is prohibited for the hospital to charge patients for PSI in Republic of Korea. Preplanning and design processes were conducted using previously validated commercial software from Materialise [14, 21], and a cost-effective 3D printer allowed us to perform this research [6]. In the design of the PSI, 3D reconstruction of the bone is a key step; thus this interobserver study was completed by two observers (Kyoung-Tak Kang, Juhyun Son) using a rule-based protocol suggested by Koo et al. for cartilage reconstruction using MRI scans [22].

In this study, the difference between CT and MRI scans was analyzed first. There is a lack of soft tissue modeling in the models based on CT scans; thus the contact surface was considered to be bone [23]. Even for a surgeon who has already experienced 30 cases of PSI-TKA, a surgical guide with only several points of contact on the bony surface without considering the cartilage thickness was not ideal. Similar uncertainties from CT scans have been reported in previous studies as well [24, 25].

The required scan time for MRI with 1 mm slice thickness is about 70% more than that of MRI with 2 mm slice thickness. There are many motion artifacts disturbing the design of PSI guides in the 1 mm slice MRI scan, especially in elderly patients [8]. For this reason, MRI-based PSI guide designs were modified with respect to the quality of the MRI scans. The first criterion is the existence of motion artifacts. Asada et al. reported that 35% of patients from their study were dropped due to motion artifacts in the MRI-based PSI guide [8]. In our study, the importance of the elimination of motion artifacts during MRI scanning was also emphasized. In other words, small increments in slice thickness are not always accepted as good data. The reasons that implant size and alignment may vary intraoperatively were that PSI guide was designed and manufactured without considering quality of medical image and having unstable contact.

For CT based PSI guides, it did not provide a good stability due to the absence of soft tissue such as articular cartilage and contact using spike for the gap between guide and bone. MRI based PSI guides designed without regard to patient's motion artifact could lead to its instability in contact with bony surface. For MRI image with motion artifact, it was found from group 1 that it might lead to the more inaccurate result if full contact PSI design is applied. If there is uncertainty in cartilage regions due to motion artifact in MRI, it was found that tolerance in design may produce better stability in contact of PSI guide with bone. Therefore, 2 mm slice thickness conditions were used in the group 2 and group 3 designs.

For motion artifacts in group 2, the areas with cartilage were considered contact surfaces, and, if not, tolerance was applied in the design. If there were no motion artifacts, the PSI design was shape-matched following the articular surface. We developed the optimal design for PSI through the learning curve related to cases with motion artifacts. PSI design with only anterior and distal contact regions in group 2 constrained flexion and extension. The current Signature PSI guides which were similarly designed from other companies could not tightly hold internal-external rotation. Therefore, we considered the optimal design to overcome this problem by holding flexion-extension and internal-external rotation fixed. Throughout such modifications, not only translation but also rotational stability was improved during guide drilling. Throughout the process from group 1 to group 3, the intraoperative changes in alignment and implant sizing diminished.

Bone resection was measured with a 3D scanner, instead of a 2D micrometer, to obtain more precise qualitative measurements. The resection cutting results reflect how well PSI-TKA surgery was completed after preoperative planning. To our knowledge, this is the first research paper to evaluate the design of PSI guides with respect to their contact stability and relation to 3D medical images. The overall discrepancy was small for the resection thicknesses compared to the preplanned results (groups 2-3). Thus, we are satisfied with the PSI that were planned. However, it is worth noting that greater variation was noted in the distal femoral resections, which were found to be decreased in the optimal design. The significant finding in this study was that group 3 with motion artifacts and full contact design and group 2 without motion artifacts had similar discrepancies between the predicted and actual resection. In other words, design improvement may compensate for the negative effect of motion artifacts on MRI quality.

A complete patient-specific system can potentially shorten operative time, setup time, operating room space, and hospital space. Setup time is shortened by eliminating the need to bring and open multiple instrumentation sets in the operating room. Procedural time can also be reduced in the complete patient-specific system that includes all instrumentation and a patient-specific implant by eliminating several time-consuming steps. When the instrumentation and implants are completely patient-specific, the implant sizing, rotation, and positional decisions are predetermined. These implant attributes either can be based on a standard set of design rules or could be customized according to surgeon preference.

There were limitations to our study. First, this current study did not assess final implant positioning, which will be investigated in future research. Second, a single experienced surgeon made all the preplanning and intraoperative decisions concerning changes to alignment and implant sizing. This is not representative of the decisions of a low-volume or inexperienced knee surgeon using this technology. A future study with multiple surgeons would provide a more comprehensive representation of this technology based on surgeon experience. One surgeon working on the planning by himself could reduce confusion, compared to working with multiple surgeons [10]. Finally, this study focused on intraoperative validation of patient-specific instruments and comparison of immediate postoperative radiographic outcomes with conventional TKA, without encompassing other important parameters, such as functional improvement, patient satisfaction, longevity, or cost-effectiveness.

## 5. Conclusion

In conclusion, the optimum PSI design for stability improvement was suggested. In terms of 3D image scanning time required, this would be beneficial to the hospital and also to the patients. The approach outlined introduces a generic product in addition to the commercial PSI systems offered by other manufacturers. In the future, various PSI design methods should be evaluated for variable conditions ranging from different MRI systems to patient anatomy.

## Figures and Tables

**Table 1:** Summary of demographics used in this study.

Demographic	
Number of TKAs	120
Average age years (±SD)	70.9 (±9.2)
Male : female	23 : 97
Left : right	53 : 67
BMI average (kg/m^2^)	27.4

BMI: body mass index.

**Table 2:** Intraoperative changes made to the femoral components in 120 PSI.

Femur	Change made	Group I		Group II		Group III	
		(*n* = 20)		(*n* = 50)		(*n* = 50)	
		Number	Percentage	Number	Percentage	Number	Percentage
Size	Up/down	7	35%	5	10%	0	0%

Resection	Proximal	18	90%	16	32%	1	2%
	Distal	11	55%	9	18%	1	2%
	Varus	3	15%	1	2%	0	0%
	Valgus	1	5%	0	0%	0	0%

4 : 1 block	External rotation	3	15%	2	4%	0	0%
	Internal rotation	3	15%	4	16%	1	2%
	Anterior	2	10%	3	12%	0	0%
	Posterior	1	5%	2	8%	0	0%

**Table 3:** Differences between planned bone resections and bone resections recorded from the PSI intraoperatively (mm).

		Group II			Group III		
		MRI 2 mm slice thickness (w/motion artifacts) & offset design (*n* = 21)	MRI 2 mm slice thickness (w/o motion artifacts) & full contact design (*n* = 29)	Ave.	MRI 2 mm slice thickness (w/motion artifacts), offset & optimized design (*n* = 18)	MRI 2 mm slice thickness (w/o motion artifacts), full contact & optimized design (*n* = 32)	Ave.
Distal	*M* (±SD)	0.56 (±0.12)	0.28 (±0.04)	0.44 (±0.17)	0.26 (±0.04)	0.16 (±0.04)	0.21 (±0.06)
	*L* (±SD)	0.46 (±0.09)	0.18 (±0.03)	0.32 (±0.15)	0.14 (±0.03)	0.12 (±0.02)	0.13 (±0.03)

Posterior	*M* (±SD)	0.34 (±0.07)	0.23 (±0.03)	0.27 (±0.08)	0.20 (±0.03)	0.18 (±0.02)	0.19 (±0.02)
	*L* (±SD)	0.19 (±0.03)	0.17 (±0.01)	0.18 (±0.02)	0.14 (±0.02)	0.12 (±0.01)	0.13 (±0.01)

## References

[1] Fehring T. K., Odum S. M., Troyer J. L., Iorio R., Kurtz S. M., Lau E. C. (2010). Joint replacement access in 2016: a supply side crisis. *Journal of Arthroplasty*.

[2] Lombardi A. V., Berend K. R., Adams J. B. (2008). Patient-specific approach in total knee arthroplasty. *Orthopedics*.

[3] Ng V. Y., DeClaire J. H., Berend K. R., Gulick B. C., Lombardi A. V. (2012). Improved accuracy of alignment with patient-specific positioning guides compared with manual instrumentation in TKA. *Clinical Orthopaedics and Related Research*.

[4] Howell S. M., Hodapp E. E., Kuznik K., Hull M. L. (2009). In vivo adduction and reverse axial rotation (external) of the tibial component can be minimized. *Orthopedics*.

[5] Nunley R. M., Ellison B. S., Ruh E. L. (2012). Are patient-specific cutting blocks cost-effective for total knee arthroplasty?. *Clinical Orthopaedics and Related Research*.

[6] Krishnan S. P., Dawood A., Richards R., Henckel J., Hart A. J. (2012). A review of rapid prototyped surgical guides for patient-specific total knee replacement. *Journal of Bone and Joint Surgery B*.

[7] White D., Chelule K. L., Seedhom B. B. (2008). Accuracy of MRI vs CT imaging with particular reference to patient specific templates for total knee replacement surgery. *International Journal of Medical Robotics and Computer Assisted Surgery*.

[8] Asada S., Mori S., Matsushita T., Nakagawa K., Tsukamoto I., Akagi M. (2014). Comparison of MRI- and CT-based patient-specific guides for total knee arthroplasty. *Knee*.

[9] Silva A., Sampaio R., Pinto E. (2014). Patient-specific instrumentation improves tibial component rotation in TKA. *Knee Surgery, Sports Traumatology, Arthroscopy*.

[10] Stronach B. M., Pelt C. E., Erickson J., Peters C. L. (2013). Patient-specific total knee arthroplasty required frequent surgeon-directed changes. *Clinical Orthopaedics and Related Research*.

[11] Winder J., Bibb R. (2005). Medical rapid prototyping technologies: state of the art and current limitations for application in oral and maxillofacial surgery. *Journal of Oral and Maxillofacial Surgery*.

[12] Joffe J. M., Nicoll S. R., Richards R., Linney A. D., Harris M. (1999). Validation of computer-assisted manufacture of titanium plates for cranioplasty. *International Journal of Oral and Maxillofacial Surgery*.

[13] Fehring T. K., Odum S., Griffin W. L., Mason J. B., Nadaud M. (2001). Early failures in total knee arthroplasty. *Clinical Orthopaedics and Related Research*.

[14] Hafez M. A., Chelule K. L., Seedhom B. B., Sherman K. P. (2006). Computer-assisted total knee arthroplasty using patient-specific templating. *Clinical Orthopaedics and Related Research*.

[15] Werner F. W., Ayers D. C., Maletsky L. P., Rullkoetter P. J. (2005). The effect of valgus/varus malalignment on load distribution in total knee replacements. *Journal of Biomechanics*.

[16] Bauwens K., Matthes G., Wich M. (2007). Navigated total knee replacement: a meta-analysis. *The Journal of Bone & Joint Surgery—American Volume*.

[17] Slover J. D., Tosteson A. N. A., Bozic K. J., Rubash H. E., Malchau H. (2008). Impact of hospital volume on the economic value of computer navigation for total knee replacement. *The Journal of Bone and Joint Surgery—American Volume*.

[18] Chauhan S. K., Scott R. G., Breidahl W., Beaver R. J. (2004). Computer-assisted knee arthroplasty versus a conventional jig-based technique. A randomised, prospective trial. *The Journal of Bone & Joint Surgery—British Volume*.

[19] Victor J., Dujardin J., Vandenneucker H., Arnout N., Bellemans J. (2014). Patient-specific guides do not improve accuracy in total knee arthroplasty: a prospective randomized controlled trial. *Clinical Orthopaedics and Related Research*.

[20] Hamilton W. G., Parks N. L., Saxena A. (2013). Patient-specific instrumentation does not shorten surgical time: a prospective, randomized trial. *Journal of Arthroplasty*.

[21] Brown G. A., Firoozbakhsh K., DeCoster T. A., Reyna J. R., Moneim M. (2003). Rapid prototyping: the future of trauma surgery?. *The Journal of Bone & Joint Surgery—American Volume*.

[22] Koo S., Gold G. E., Andriacchi T. P. (2005). Considerations in measuring cartilage thickness using MRI: factors influencing reproducibility and accuracy. *Osteoarthritis and Cartilage*.

[23] Tibesku C. O., Innocenti B., Wong P., Salehi A., Labey L. (2012). Can CT-based patient-matched instrumentation achieve consistent rotational alignment in knee arthroplasty?. *Archives of Orthopaedic and Trauma Surgery*.

[24] Ensini A., Timoncini A., Cenni F. (2014). Intra- and post-operative accuracy assessments of two different patient-specific instrumentation systems for total knee replacement. *Knee Surgery, Sports Traumatology, Arthroscopy*.

[25] Howell S. M., Kuznik K., Hull M. L., Siston R. A. (2008). Results of an initial experience with custom-fit positioning total knee arthroplasty in a series of 48 patients. *Orthopedics*.

